# HPLC-ESI-IT-MS/MS Analysis and Biological Activity of Triterpene Glycosides from the Colombian Marine Sponge *Ectyoplasia ferox*

**DOI:** 10.3390/md11124815

**Published:** 2013-12-02

**Authors:** Jhonny Colorado-Ríos, Diana Muñoz, Guillermo Montoya, Diana Márquez, Maria-Elena Márquez, Juan López, Alejandro Martínez

**Affiliations:** 1 Grupo de Investigación Productos Naturales Marinos, Facultad de Química Farmacéutica, Universidad de Antioquia, Calle 70 N° 52-21, Medellín, Colombia; E-Mails: dmarquez@farmacia.udea.edu.co (D.M.); amart@farmacia.udea.edu.co (A.M.); 2 Unidad de Investigación e Innovación, Humax Pharmaceutical S.A, 050010, Itagüí, Colombia; 3 Grupo de Biotecnología Animal, Universidad Nacional de Colombia, 050034, Medellín, Colombia; E-Mails: ingbiodianacaro@gmail.com (D.M.); memarque@unal.edu.co (M.-E.M.); jblopez@unal.edu.co (J.L.); 4 Grupo Natura, Facultad de Ciencias Naturales, Universidad ICESI, 760050, Cali, Colombia; E-Mail: glmontoya@icesi.edu.co

**Keywords:** *Ectyoplasia ferox*, marine sponge, triterpenoid glycosides, mass spectrometry

## Abstract

The marine sponge *Ectyoplasia ferox* produces antipredatory and allelopathic triterpenoid glycosides as part of its chemical defense repertoire against predators, competitors, and fouling organisms. These molecules are responsible for the pharmacological potential found in the glycosides present in this species. In order to observe the glycochemical diversity present in *E. ferox*, a liquid chromatography coupled to a tandem mass spectrometry approach to analyse a complex polar fraction of this marine sponge was performed. This gave valuable information for about twenty-five compounds three of which have been previously reported and another three which were found to be composed of known aglycones. Furthermore, a group of four urabosides, sharing two uncommon substitutions with carboxyl groups at C-4 on the terpenoid core, were identified by a characteristic fragmentation pattern. The oxidized aglycones present in this group of saponins can promote instability, making the purification process difficult. Cytotoxicity, cell cycle modulation, a cell cloning efficiency assay, as well as its hemolytic activity were evaluated. The cytotoxic activity was about IC_50_ 40 µg/mL on Jurkat and CHO-k_1_ cell lines without exhibiting hemolysis. Discussion on this bioactivity suggests the scanning of other biological models would be worthwhile.

## 1. Introduction

Sponges are an important source of secondary metabolites; most of them exhibit bioactivity which has been exploited for discovering potential therapeutic compounds. However, such potential has been barely investigated [1]. This chemical diversity has been associated to the lack of physical defenses, implying the development of chemical defenses to fight predators [2,3]. 

One of the most significant problems that has hindered research on secondary metabolites in sponges is their low concentration; for marine invertebrates various compounds are found at concentrations of less than 0.00001% of their body weight [4], making mandatory the use of techniques like chromatography coupled with mass spectrometry, which has proven to be a powerful analytical tool for the fast and effective investigation of complex biomolecules [5].

**Figure 1:**
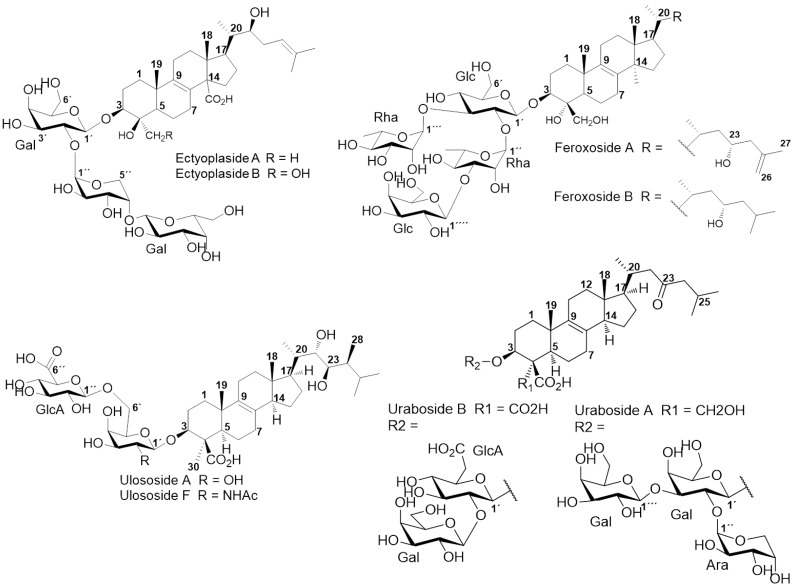
Structures of saponins from *E. ferox*.

Triterpenoid and/or steroidal saponins are glycoconjugates released by invertebrates into the marine environment such as echinoderms and soft corals, acting as cytotoxic weapons. Similarly, these glycosides have been widely reported due to their impressive diversity with respect to triterpenoid/steroid aglycones and carbohydrate moieties [6,7]. In sponges, few chemical studies emphasizing their presence have been reported, revealing special chemical features in genera such as *Erylus*, *Mycale*, *Melophlus* [6,7] and *Pandaros* species with a significant amounts of those compounds [8,9]. Other genera from the order *Astrophorida*, *Poecilosclerida*, *and Halichondrida. Ectyoplasia ferox* (Duchassaing and Michelotti, 1864) (Poecilosclerida, Raspailiidae) from the Urabá Gulf (Colombia) attracted our attention due to their triterpenoid glycosides composition and their chemical differences related to geographical and environmental factors. This was previously shown by our laboratory, where three new triterpenoid glycosides were isolated from a polar complex fraction, along with a previously reported ulososide [10]. The structures for these new compounds were clearly different from ectyoplasides and feroxosides previously reported for the same species but at another geographical location at the Northern part of the Caribbean (Figure 1). The impressive defensive, antifouling, and allelopathic biological properties of *E. ferox* among Caribbean sponges could be connected to its relevant pharmacological potential, increasing research on the chemical diversity and bioactivity of its glycosides [7,11].

A liquid chromatography/electrospray ionization tandem mass spectrometry (LC/ESI-MS*^n^*) approach was used in order to assess the molecular weight and characteristic fragmentations of aglycones and monosaccharides, allowing a rapid dereplication of the polar complex mixture from *E. ferox*. This fraction contains glycoconjugates with pharmaceutical potential, owing to its poor hemolytic activity, which is the most common side effect for this class of substances as well as being a considerable obstacle for clinical trials progress [12,13].

## 2. Results and Discussion

### 2.1. Chemical Analysis

By using the previously described approach [14], a total of twenty-five saponins were identified in a polar complex mixture from *Ectyoplasia ferox*, from which ulososides A and D, and urabosides A and B are well-known molecules (Figure 1 and Table 1). None of the other twenty-one identified compounds have been previously reported for this species. 

**Figure 2:**
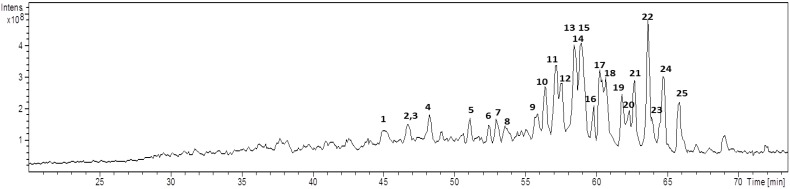
Total Ion Chromatogram of saponins from *E. ferox* acquired by LC-ESI-MS (*m*/*z*: 400–1500 Da).

Because of the high affinity of alkali cations for triterpene glycosides [15], the mass spectra of all the saponins are dominated by [M + Na]^+^ ions, improving the analysis. The full-HPLC-ESI-MS profile for the saponins mixture is shown in Figure 2. Twenty-five triterpene saponins were identified according to the characteristic fragmentation reactions observed at the corresponding mass spectra, which also enabled the identification of sequences of aglycones and monosaccharides. Regardless of the complexity of this profile, even overlapping peaks were assessed because their relative intensities were sufficient to determine each peak and its corresponding triterpenoid glycosides. Regarding the relative intensity, the saponin detected at *m*/*z* 805 (Rt: 62.2 min) as a sodium cation was highlighted as the most intense signal, however, it was not the most abundant in the sponge [10]. 

**Table 1:** Saponins found in *Ectyoplasia ferox*, with their LC/MS/MS retention times and fragment signatures.

Cmp	Rt (min)	*m*/*z* [M + Na]^+^	MW	Diagnostic ions (*m*/*z*) ^a^	Aglycone core ^b^	Glycan core
1	45.1	851	828	833, 513 [M − HexA − Hex + Na]^+^, 495 [M − HexA − Hex − H_2_O + Na]^+^, 452	ULO-A	Hex-HexA-
2	46.4	851	828	675 [M − HexA + Na]^+^, 513 [M − HexA − Hex + Na]^+^, 495 [M − HexA − Hex − H_2_O + Na]^+^, 451	ULO-A	HexA-Hex- ^c^
3	46.6	985	962	967, 923, 853 [M − Pen + Na]^+^, 791, 497 [M − Aglycone + Na]^+^, 479 [M − Aglycone − H_2_O + Na]^+^	AG-1	Pen-Hex-Hex ^d^
4	48.0	851	828	833, 801, 513 [M − HexA − Hex + Na]^+^, 495 [M − HexA − Hex − H_2_O + Na]^+^	ULO-A	HexA-Hex-
5	50.7	837	814	661 [M − HexA + Na]^+^, 675 [M − Hex + Na]^+^, 499 [M − HexA − Hex + Na]^+^, 435	AG-2	HexA, Hex ^e^
6	52.3	716	693	684, 513 [M − HexNAc + Na]^+^, 495 [M − HexNAc − H_2_O + Na]^+^, 451	ULO-A	HexNAc-
7	53.0	819	796	687 [M − pen + Na]^+^, 525 [M − Pen − Hex + Na]^+^, 479	AG-3	Pen-Hex-R
8	53.6	967	944	905 [M − CO_2_ − H_2_O + Na]^+^, 835 [M − Pen + Na]^+^, 673 [M − Pen − Hex + Na]^+^, 497 [M − Aglycone + Na]^+^	URA-B	Pen-Hex-Hex ^d^
9	54.0	951	928	819 [M − Pen + Na]^+^, 497 [M − Aglycone + Na]^+^	AG-4	Pen-Hex-Hex ^d^
10	56.1	979	956	497 [M − Aglycone + Na]^+^	AG-5	Pen-Hex-Hex ^d^
11	57.0	953	930	821 [M − Pen + Na]^+^, 791 [M − Hex + Na]^+^, 497 [M − Aglycone + Na]^+^, 479 [M − Aglycone − H_2_O + Na]^+^	URA-A	Pen-Hex-Hex ^d^
12	57.6	937	914	875 [M − CO_2_ − H_2_O + Na]^+^, 805 [M − Pen + Na]^+^, 467 [M − Aglycone + Na]^+^, 449 [M − Aglycone − H_2_O + Na]^+^	URA-B	Pen-Pen-Hex-
13	58.2	921	898	789 [M − Pen + Na]^+^, 657 [M − 2 Pen + Na]^+^, 467 [M − Aglycone + Na]^+^, 449 [M − Aglycone − H_2_O + Na]^+^	AG-4	Pen-Pen-Hex-
14	58.4	1097	1074	965 [M − Pen + Na]^+^, 921 [M − HexA + Na]^+^, 833 [M − 2 Pen + Na]^+^, 657 [M − 2 Pen − HexA + Na]^+^	AG-4	Pen-Pen-HexA-Hex-
15	58.6	1111	1088	965 [M − dHex + Na]^+^, 833 [M − dHex − Pen + Na]^+^, 657 [M − dHex − Pen − HexA + Na]^+^, 495 [M − dHex − Pen − HexA − Hex + Na]^+^	AG-4	dHex-HexA-Pen-Hex-
16	59.7	937	914	805 [M − Pen + Na]^+^, 511 [M − 2 Pen − Hex + Na]^+^, 493 [M − 2 Pen − Hex − H_2_O + Na]^+^, 467 [M − Aglycone + Na]^+^, 431	URA-B	Pen-Pen-Hex-
17	60.1	965	942	833 [M − Pen + Na]^+^, 789 [M − Pen − COO + Na]^+^, 657 [M − Pen − HexA + Na]^+^	AG-4	Pen-HexA-Hex-
18	60.3	849	826	805 [M − CO_2_ + Na]^+^, 787 [M − COO − H_2_O + Na]^+^, 449 [M − COO − HexA − Hex − H_2_O + Na]^+^	URA-B	Hex-HexA ^f^
19	61.6	803	780	641 [M − Hex + Na]^+^, 465 [M − Hex − HexA + Na]^+^	AG-6	Hex-HexA ^f^
20	62.2	805	782	743, 673 [M − Pen + Na]^+^, 611 [M − HexA − H_2_O + Na]^+^	URA-A	Pen-HexA ^e^
21	62.7	803	780	641 [M − Hex + Na]^+^, 627 [M − HexA + Na]^+^, 465 [M − Hex − HexA + Na]^+^	AG-4	Hex-HexA ^f^
22	63.5	805	782	643 [M − Hex + Na]^+^, 467 [M − Hex − HexA + Na]^+^	AG-7	Hex-HexA ^f^
23	63.9	817	794	641 [M − HexA + Na]^+^, 479 [M − HexA − Hex + Na]^+^	AG-8	HexA-Hex ^c^
24	64.5	761	738	629 [M − Pen + Na]^+^, 467 [M − Pen − Hex + Na]^+^	AG-7	Pen-Hex-
25	65.8	787	764	655 [M − Pen + Na]^+^, 479 [M − Pen − HexA + Na]^+^	AG-8	Pen-HexA-

^a^ Monosaccharides were identified by residues masses of 162 for hexoses (Hex), 176 for hexuronic acids (HexA), 132 for pentoses (Pen) and 203 for *N*-acetylhexosamines (HexNAc); ^b^ Aglycone cores are directly associated with the mass of our previous report as ULO-A: Aglycone from ulososide A (490 Da); URA-A: Aglycone from uraboside A (474 Da); URA-B: Aglycone from uraboside B (488 Da); and other new aglycones with a specific *m*/*z* as AG-1 (506 Da), AG-2 (476 Da), AG-3 (502 Da), AG-4 (472 Da), AG-5 (500 Da), AG-6 (442 Da), AG-7 (444 Da) and AG-8 (456 Da); ^c^ Glycan core could be the same for ulososide A such as *O*-[β-d-Glucuronopyranosyl-(1→6)-β-d-glucopyranoside]; ^d^ Glycan core could be the same for uraboside A such as *O*-[β-d-Arabinopyranosyl-(1′′→2′)-(β-d-galactopyranosyl-(1′′′→3′))-β-d-galactopyranoside]; ^e^ Bidesmoside-like fragmentation; ^f^ Glycan core could be the same for uraboside B such as *O*-[β*-*d-Galactopyranosyl-(1→2)-β-d-glucopyranosiduronic acid].

**Figure 3:**
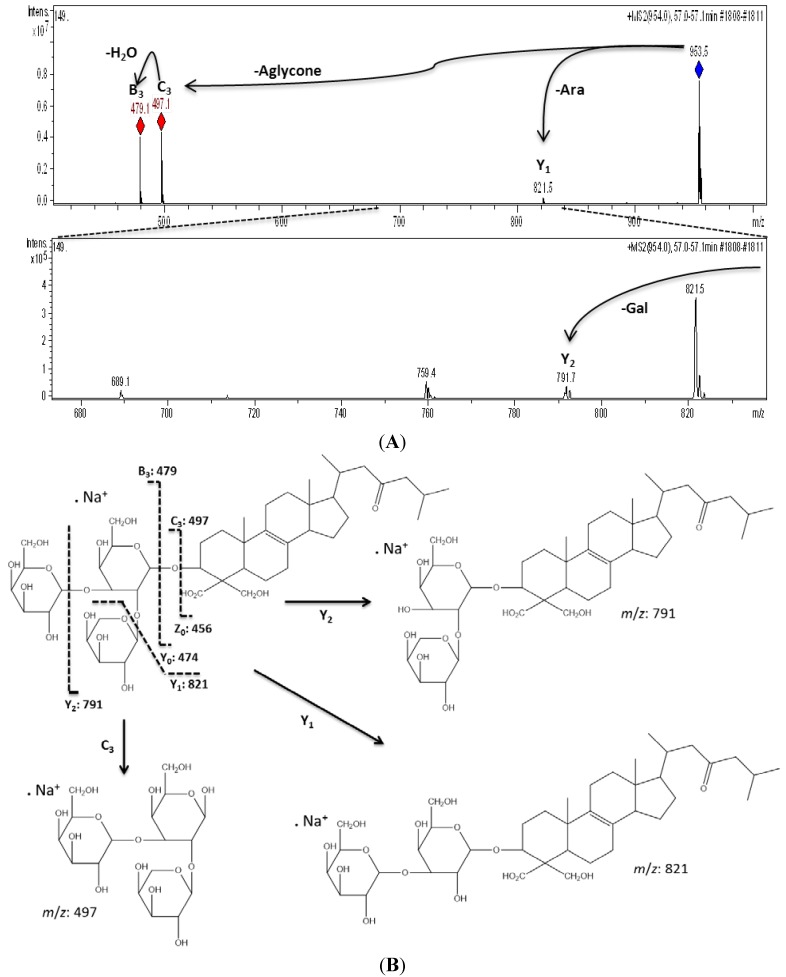
Collision-induced fragmentation pattern for uraboside A (compound **11**).

Campagnuolo *et al*. [16] and Cafieri *et al*. [17], described four different saponins for *E. ferox* such as: Ectyoplaside A ([M + Na]^+^: *m*/*z* 953), Ectyoplaside B ([M + Na]^+^: *m*/*z* 969), Feroxoside A ([M + Na]^+^: *m*/*z* 1099) and Feroxoside B ([M + Na]^+^: *m*/*z* 1101). Those authors described the molecular structures of these saponins; however, it was also possible to determine in a previous report strong structural differences between triterpenoid skeletons of saponins from the Colombian sponge compared to that initially discovered from the Bahamas [10]. The saponins reported were: ulososide A ([M + Na]^+^: *m*/*z* 851), ulososide F ([M + Na]^+^: *m*/*z* 892), uraboside A ([M + Na]^+^: *m*/*z* 953) and uraboside B ([M + Na]^+^: *m*/*z* 849). Considering only the *m*/*z* value of the detected ions by means of the first-order ESI mass spectra from the HPLC-MS assay (Figure 2), three among these were also identified with the current experiment at *m*/*z* 851, 953 and 849. To further validate if detected signals at the first order ESI mass spectra correspond to saponin ions, ESI/MS/MS analysis was carried out for each one. As a typical example, the ESI/MS/MS mass spectrum for the ion detected at *m*/*z* 953 (**11**) is shown in Figure 3A. These MS^2^ and MS^3^ spectra allowed construcion of a collision-induced fragmentation pattern of the parent ion and resulted exclusively in fragment ions originating from the cleavage of one (B-, C-, Y-, and Z-type ions according to the nomenclature of Domon and Costello [18]) and two glycosidic bonds (internal cleavage ions). The B, C and Y cleavage ions clearly show that the glycan is a branched trisaccharide of two hexoses and one pentose. Therefore, the CID spectrum is consistent with the structure of uraboside A, hence, the proposed fragmentation pathway shown in Figure 3B could be established. After collisional activation, *m*/*z* 953 cations were submitted to two competitive dissociation pathways. First, as described in Figure 3A, simultaneous losses of galactose (Gal) and arabinose (Ara) residues afforded product ions detected at 791 (Y_2_) and 821 (Y_1_), respectively. Then, the fragmentation of the parent ions could also be initiated by the loss of the aglycone moiety, generating *m*/*z* 497 (C_3_) cations (Figure 3B).

**Figure 4:**
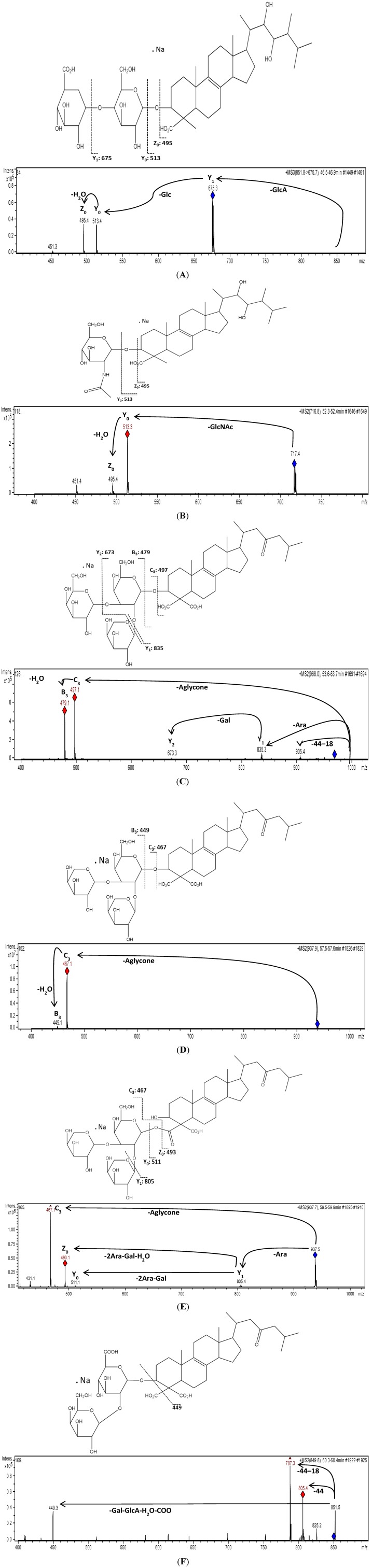
ESI-IT-MS*^n^* mass spectrum analysis and presumptive molecular structures of selected saponins detected in a fraction of *Ectyoplasia ferox*. Graphical representations of ions generated by sodium cations in positive acquisition mode accord with the Domon and Costello nomenclature [18]. (**A**) shows the MS^3^ spectra of compound **2** (ulososide A); (**B**) compound **6** (ulososide D); (**C**) compound **8**; (**D**) compound **12**; (**E**) compound **16** and (**F**) compound **18** (uraboside B).

Similar fragmentation patterns were obtained for the detected ions by ESI ionization of the total fraction (Table 1). This approach for molecular structure identification using fragmentation patterns derived from MS^2^ and MS^3^ measurements indicated that the detected ions at *m*/*z* 851 (**2**), 716 (**6**) and 849 (**18**) are consistent with ulososide A, uloloside D and uraboside B, respectively (Figure 4), in comparison with previous reports for these compounds [10,19,20]. The precursor ion at *m*/*z* 851 was detected at three different retention times (45.1, 46.4 and 48.0 min), however, the identification of ulososide A as the peak at 46.4 min, did not represent a problem due to its fragmentation pattern (Figure 4A). The fragmentation of compound **2** (*m*/*z* 851) resulted in *m*/*z* 675, corresponding to the [M + Na − 176]^+^ ion, which is consistent with the loss of HexA. Then a sequential ion cleavage at *m*/*z* 513 was consistent with the [M + Na − 176 − 162]^+^ ion suggesting a hexuronic acid linked to a hexose according to its GlcA-Glc- as in ulososide A. This compound, together with **6** (*m*/*z* 716), produced the same ions cleavage for the aglycone at *m*/*z* 495 (Z_0_) and *m*/*z* 513 (Y_0_), therefore, the same aglycone was outlined. Compound **6** corresponded to the structure of a previously reported ulososide D with a GlcNac linked at the C-3 of the aglycone [20] according to the [M + Na − 203]^+^ ion at *m*/*z* 513 (Figure 4B). 

Based on the recorded MS^2^ spectra, three additional urabosides were identified at *m*/*z* 967 (**8**), 937 (**12**) and 937 (**16**) respectively (Figure 4C–E). All these saponins were quite easily recognized upon MS^2^ measurement since their CID spectra shared common key signals and losses which allowed the establishment of carbohydrate moieties besides the same aglycone mass of the uraboside B. A common feature of these urabosides is their uncommon substitution pattern at C-4 with carboxyl groups at the terpenoid core. This aglycone has never been discovered in other molecules. Therefore, this feature was identified as characteristic ions caused by CID, corresponding to the loss of one carboxyl group [M − 44 + Na]^+^ and followed by the loss of a water molecule [M − 44 − 18 + Na]^+^ (Figure 4F). Due to the absence of this fragmentation pattern in compound **16** we suggest that the carbohydrate core is linked to the aglycone through one of the carboxyl groups at C-4 (Figure 4E), being a positional isomer of compound **12** (Figure 4D). 

The complete LC/MS/MS analysis for the polar complex mixture of *E. ferox* is summarized in Table 1. This table shows that at least 25 saponins have been detected and their compositions proposed. However, other congeners are present in non-isolable quantities and need further investigation by other techniques and approaches. So far, retention time is the only feature that enables differentiation between some isomeric saponins, even if the mass spectra give some clues for aglycone-glycan assembly checking. 

Fragmentation pathways for all the compounds showed a group of saponins comprising known and novel aglycones and glycan cores present in this species. According to the discussion above, fragmentation of several compounds gives [M − aglycone + Na] ions at *m*/*z* 497 followed by [M − aglycone − H_2_O + Na] ions at *m*/*z* 479 when the carbohydrate core was [β-d-Arabinopyranosyl-(1′′→2′)-(β-d-galactopyranosyl-(1′′′→3′))-β-d-galactopyranoside] as in uraboside A (compound **11**). Following this pattern, compounds **3**, **8**, **9** and **10** share this common carbohydrate core. Compounds **3**, **9** and **10** showed neutral losses of 506 u, 472 u and 500 u, respectively, suggesting marked differences among their aglycones. 

The identification of another set of saponins with the *m*/*z* 513 signature (like compounds **1**, **2**, **4** and **6**), allowed the addition of two new molecules to the list of ulososides reported for *E. ferox*, from which, **2** and **6** were consistent with the structures of ulososide A and D, respectively (Figure 4A,B). In fact, related to previous reports for the other saponins, compounds **1** and **4** could be named perfectly as new ulososides (Table 1). 

For *E. ferox* saponins, a clear relationship between retention time and the saccharide and aglycone moieties modifications was not observed, perhaps, due to the high number of aglycones present at the same fraction (11 aglycone cores) from which only three of them are associated to known saponins [10]. For some compounds with the same carbohydrate core, the aglycones modification had small effect on the retention time; whereas for others, an exchange of the aglycone shifts significantly the retention time. Similar results were observed for compounds **8**–**11**, sharing part of their saccharide composition, branching and sequence but with varying aglycones. Due to the similar polarity of URA-B, AG-4, AG-5 and URA-A aglycones, those compounds eluted at 53, 54, 56 and 57 min, respectively, and were separated with poor resolution, as well as compounds **13**–**15** bearing the same aglycone but different carbohydrate moieties which co-elute at 58 min as two unresolved peaks (Figure 2). 

Although we show a representative description of the complexity of saponins from *E. ferox*, mass spectrometry by itself is not enough in order to obtain further information about the structures. Nevertheless, it allows a quick and straightforward characterization of the fraction composition by the presence of characteristic ions in the MS^2^ and MS^3^ spectra.

### 2.2. Biological Analysis

#### 2.2.1. Cytotoxicity

Cytotoxicity of the saponins mixture from *E. ferox* was assessed by multiple *in vitro* bioassays with the human cancer cell line (Jurkat) and the hamster cell line (CHO-k_1_). First of all, viable cells were tested with the trypan blue exclusion assay and also hemolytic activity was observed, exploring the cytotoxicity after by MTT assay [21]. As shown in Table 2, the viability of CHO-k_1_ and Jurkat cells was quantified in culture with a mixture of saponins during 24 h by trypan blue assay. The trypan blue-exclusion assay showed a significant decrease in cell viability after treatment with saponins for Jurkat and CHO-k_1_ cells. The observed cytotoxicity did not involve hemolytic activity in human erythrocytes (at least after one hour of treatment). These results indicate that cytotoxic activity of this fraction did not involve a lytic mechanism. It was also observed that the effect was stronger in CHO-k_1_ cells at 50 µg/mL than for Jurkat cells under the same treatment. 

**Table 2:** Cell viability and hemolytic activity for saponins from *E. ferox*. All data represent average ± SEM of fraction of cell viability, which was derived from two and three replicates, respectively. * *p* <0.05 when comparison is made against solvent (PBS) and negative control (cells without treatment).

Treatment µg/mL	CHO cell line	Jurkat cell line	Hemolytic activity
Solvent (PBS 5%–10%)	0.97 ± 0.01	0.98 ± 0.01	0.31 ± 0.03
0	0.90 ± 0.02	0.97 ± 0.00	0.00 ± 0.00
25	0.9 ± 0.06	0.83 ± 0.15	-
50	0.04 ± 0.01	0.35 ± 0.01	0.43 ± 0.16
100	0,00 ± 0.00	0.00 ± 0.00	0.67 ± 0.27
200	-	-	0.36 ± 0.10

**Figure 5:**
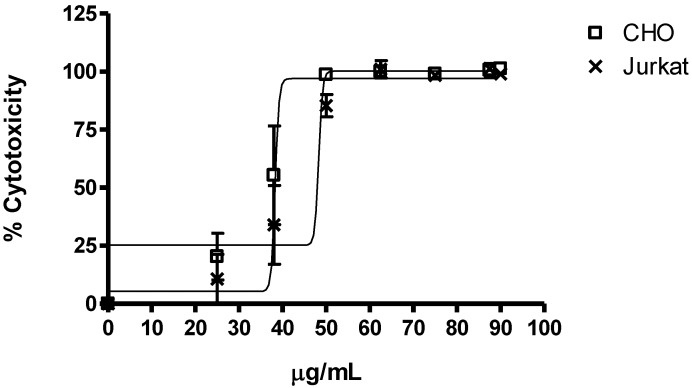
Averages of cytotoxicity values of the saponins mixture from *E. ferox* on Jurkat and CHO-k_1_ cell lines were determined by MTT assay. Data came from the mean ± SEM of percentage of cell cytotoxicity which was derived from three replicates.

The saponins mixture showed significant cytotoxic activity on Jurkat and CHO-k_1_ cell lines as shown by the MTT test (Figure 5). This assay showed significant differences between the activity of the concentration of each saponin *versus* the negative control (cells without treatment) in CHO-k_1_ and Jurkat cells (*p*-Value < 0.0001); displaying an IC_50_ estimated for CHO-k_1_ cells at 38 µg/mL, as well as for Jurkat cells at 48 µg/mL. Analysis of the values in Figure 4 indicated that the responses to saponins were similar, indicating less variation in the sensitivity between cells to the saponins fraction. In addition, these results show that the saponins fraction has a strong cytotoxic profile for two cell lines (as observed with the 25 µg/mL concentration). 

#### 2.2.2. Antiproliferative and Cell Cycle Progression

As soon as the cytotoxic activity of the saponins fraction was demonstrated, its antiproliferative activity was assessed. Two classical cytogenetic assays were performed. Cell division ability was evaluated by the cloning efficiency assay, and the mitotic index (MI) was a sensitive indicator for cell cycle and growth progression [22]. The results showed that the saponins fraction slightly affected the cloning efficiency and proliferative capacity of the two cell lines (Table 3). 

**Table 3:** Average of the absolute and relative cloning efficiency assays in CHO-k_1_ and Jurkat cell lines with 95% LDS intervals. Data came from the mean ± SEM of percentage of cytotoxicity which were derived from three replicates.

Treatment µg/mL	*Absolute cloning efficiency*			*Relative cloning efficiency*		
	Mean ± SEM × 100	Low limit	High limit	Mean ± SEM × 100	Low limit	High limit
**CHO cell line**						
0	1.13 ± 0.15	0.96	1.31			
Solvent (PBS 10%)	0.84 ± 0.08	0.67	1.02	0.77 ± 0.17	0.57	0.96
7.5	0.98 ± 0.01	0.81	1.15	0.87 ± 0.07	0.68	1.06
15	0.68 ± 0.09	0.51	0.86	0.62 ± 0.02	0.42	0.81
30	0.55 ± 0.09	0.38	0.73	0.48 ± 0.03	0.29	0.68
**Jurkat cell line**						
0	2,46 ± 0.2	1.43	3.48			
Solvent (PBS 10%)	1.53 ± 1.35	0.51	2.55	0.67 ± 0.6	0.07	1.27
8.6	2.87 ± 0.44	1.84	3.89	1.16 ± 0.09	0.56	1.76
17	1.56 ± 0.17	0.54	2.58	0.63 ± 0.02	0.03	1.23
26	0.19 ± 0.01	−0.84	1.21	0.08 ± 0.0	−0.52	0.67

**Figure 6:**
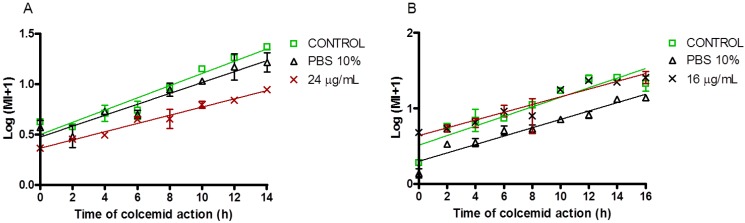
Mitotic Index for the CHO-k_1_ and Jurkat cell lines exposed to the saponins mixture from *E. ferox*. CHO-k_1_ (**A**) and Jurkat (**B**) cell lines were incubated in absence (control) or presence of IC25 concentration of the saponins mixture and solvent (PBS 10%) for 24 h. The data came from the mean ± SEM of two replicates.

The saponins fraction showed a strong cytotoxicity and also a slightly antiproliferative activity. Maybe the strong effect of this fraction on cell viability involves other molecular mechanism affecting cell cycle progression. In order to respond to this question, we performed an analysis of curves for the mitotic index and we observed that the saponins mixture affected the cell cycle progression of the CHO-k_1_ cell line but not the Jurkat cell line one (Figure 6). 

The mitotic index for CHO-k_1_ cells was visibly affected by the long treatment with the fraction during 14 h of culture. This concords with the stronger cytotoxic profile observed on CHO-k_1_ cells (compared to Jurkat cells). For that, the fraction could induce a cell cycle arrest as a first response to the cytotoxic activity. The difference with Jurkat cells is low in terms of the curve slope. The cell membrane of Jurkat cells is more resistant to the toxicity of the saponins [23], which agrees with the declined cell viability observed on the trypan blue exclusion assay. 

These results allow us to assume that the saponins fraction induces a stress response in the CHO-k_1_ and Jurkat cell lines. Apoptosis and autophagy are two common cell death mechanisms involved in the stress response of cells, and have been associated with several cytotoxic agents (like reactive oxygen species production [24]). CHO-k_1_ cells were more sensitive to the toxic effect of compounds of saponins which would cause the observed cell cycle arrest in the G1/S phase [25]. Jurkat cells showed an apparent resistance to the cytotoxic effect but their metabolic function was affected. 

## 3. Experimental Section

### 3.1. Biological Material

The marine sponge was collected off the Caribbean Sea, Colombia, in October 2008 by SCUBA diving (Urabá Gulf 8°40′14′′ N, 77°21′28′′ W). A voucher specimen (INV-POR 0335) identified by Sven Zea, was deposited in the sponge collection of the Museo de Historia Natural Marina de Colombia, Invemar. The sponge was kept frozen from collection until the extraction process.

### 3.2. Extraction of Saponins

The frozen sponge (350 g wet) was cut into pieces of about 1 cm^3^ and extracted with 1:1 MeOH/CH_2_Cl_2_ (600 mL, 24 h) at room temperature yielding 3.6 g of crude extract after solvent evaporation. The crude extract was fractionated by RP-C_18_ column chromatography (Lichroprep^®^ RP-18 40–63 µm, Merck, Darmstadt, Germany). It was eluted with a decreasing polarity gradient of H_2_O/MeOH from 70:30 to 0:100, then MeOH/CH_2_Cl_2_ from 100:0 to 0:100. The first fraction (175.5 mg), eluted after addition of the second eluent (H_2_O/MeOH 50:50), showed to be the more polar complex mixture when it was submitted to HPLC-MS assays. 

### 3.3. Liquid Chromatography/Electrospray Ionization Mass Spectrometry

HPLC separations were performed on an Agilent 1200 series system using a C_18_ reversed-phase analytical column (250 mm × 4.6 mm, 5 µm; Zorbax SB) at 35 °C. The mobile phase (water/acetonitrile) comprised 15% acetonitrile for 10 min, 15% to 35% acetonitrile from 10 to 50 min, and 35% to 50% acetonitrile from 50 to 60 min. Formic acid 0.1% (v/v) was added to the mobile phase. The flow rate was 0.8 mL/min. System control and data evaluation were carried out using the Agilent LC-MSD Chemstation software package. ESI-MS was performed on an Agilent LC-MSD ion trap mass spectrometer. The LC/ESIMS conditions were as follows: nitrogen was the nebulizer and drying gas at a pressure of 75 psi and a flow rate of 12 L/min, respectively. The temperature of the drying gas was set to 350 °C, the capillary voltage was set to ±4.5 kV and the fragmentor voltage was set at 200 V. Mass spectra were obtained over a range of *m*/*z* 400–1500 operating in positive ion mode.

### 3.4. Cell Cultures

Jurkat and CHO-k_1_ cells were cultured at 37 °C in a humidified 5% CO_2_ atmosphere in 24 cm^2^ cell culture flasks in RPMI 1640 medium supplemented with 5% heat inactivated fetal bovine serum, 2 mM l-glutamine, free of antibiotics.

### 3.5. MTT Test

Solutions at final concentrations ranging from 10 to 100 µg/mL were added to 100 µL of the CHO-k_1_ and Jurkat cells suspension (1.0 × 10^4^/well for Jurkat cells and 8.0 × 10^3^/well for CHO-k_1_ cells in RPMI 1640 medium with 5% of FBS) onto wells. Cells were treated with each sample for 24 h at 37 °C in 5% CO_2_. After treatment, the viability of cells was evaluated by the MTT assay. MTT reagent was added to each plate, and after 4 h incubation, 100 µL of acidic isopropyl alcohol mixture (50 mL Triton X-100, 4 mL of 30% HCl and 446 mL of isopropyl alcohol) was added to dissolve the water-insoluble formazan salt overnight. The OD_570_ nm absorbance was measured. These mixtures were tested three times and unexposed cells were regarded as 100% viable [21].

### 3.6. Cell Cloning Efficiency Assay

The cell cloning efficiency assay was performed as in Freshney, 2010 [22], with some modifications. The saponins fraction at a final concentrations below the IC_50_ (see Results and Discussion) was added to 1 mL of CHO-k_1_ and Jurkat cells (20,000 cells/well for Jurkat cells and, 50,000 cells/well for CHO-k_1_ cells in RPMI 1640 medium with 5% of FBS) onto wells. Cells were treated with each sample for 24 h at 37 °C in 5% CO_2_. The cells were trypsinized to produce a single-cell suspension. CHO-k_1_ cells were seeded immediately (6 well microplates) using a 2000 cells/mL dilution, at a concentration that gives 200 colonies per well. Jurkat cells were diluted until 4 × 10^4^ cells/mL in agar 0.3% (Sigma, St. Louis, MO, USA) (Agar 1.2%, ultrapure H_2_O, and RMPI 2X, 1:1:2). This mixture was dispersed evenly in each of twelve wells of a microplate, previously prepared with an agar 1.2% layer (Agar, RPMI 1640 2X, 55 °C). Cells were incubated at 37 °C in 5% CO_2_ for 6 days (CHO-k_1_ cell line) and 5 days (Jurkat cell line). The controls were without treatment, or treated with Mitomycin C (10 µg/mL, Sigma), or solvent (PBS 10%). Each experiment was done per duplicate and the absolute and relative cloning efficiency was calculated as: ACE = (number of colonies/number of seeded cells) × 100; RCE = (ACE treatment/ACE control cells). 

### 3.7. Mitotic Index

The saponins fraction at final concentrations below IC_50_ (24 and 16 µg/mL for CHO-k_1_ and Jurkat cells, respectively) was added to 2.5 mL of the cells (20,000 cells/well for Jurkat and CHO-k_1_ cells in RPMI 1640 medium with 5% of FBS) onto 6 wells microplates. Cells were treated with each sample for 24 h at 37 °C in 5% CO_2_. Later, fresh medium was added and a colcemid solution was applied to each well (10 µg/mL, G&M). Each sample was collected and processed every two hours, until 14 (CHO-k_1_ cell line) or 16 h (Jurkat cell line). The classical cytogenetic Giemsa staining method was used to obtain the metaphases dying [22]. The mitotic index was calculated as: MI = number of metaphases/total number of cells. The total number was 1000 cells.

### 3.8. Hemolysis Assay

The method was adapted from van Duick *et al.* [26] and from Taniyama *et al.* [27]. Blood was obtained from healthy young male donors. The red blood cells (RBCs) were washed three times and re-suspended in sterile RPMI 1640 medium (S&A) PBS to give about 15 × 10^6^ cells per mL and be further processed. The erythrocytes were incubated with compounds in a range 100–200 µM for 1 h at 37 °C. After centrifugation of the non hemolysed erythrocytes (800 rpm, 5 min), the absorbance of the released hemoglobin in the wavelength 540 nm was measured. The percentage of hemolysis was determined by comparing the absorbance of hemoglobin at 540 nm released from the RBCs in the presence of each compound. The positive control (100% hemolysis) was determined by the amount of hemoglobin released from 15 *×* 10^6^ RBCs after 1 h of incubation with 0.1% of Tween-20.

All statistic data were analyzed with GraphPad Prism Software. 

## 4. Conclusions

Extensive data analysis through liquid chromatography coupled with mass spectrometry enabled the identification of 21 additional saponins in the marine sponge *Ectyoplasia ferox* from the Urabá Gulf-Colombia. Considerable fragmentation information was acquired, which can be very useful for the elucidation of the structure of known and unknown triterpenoid glycosides. On *E. ferox*, these compounds were shown to be very diverse according to the structures of both aglycones and carbohydrate moieties and also by comparing with other glycosides from individual species, even from marine sponges. The fragmentation mechanisms together with strict NMR analysis (shown in a previous study for this species) resulted in an easier and correct representation of twenty-five structures. The chemical diversity of the saponins from *E. ferox* illustrate that this species develops a very complex metabolomic activity which is probably determined by microorganism-sponge interactions and/or multiple defensive roles as proposed by other authors.

The fraction of saponins promoted a reduction on the viability and the cell cycle in CHO-k_1_ and Jurkat cells, probably at the G1/S transition step. We suggest that these cells died by an apoptotic process (at least partially which must be verified). Further studies should be conducted to clarify the responsible molecules and the mechanism of action. These results are sufficient to conclude that the saponins present as a fraction in *E. ferox* have a valuable cytotoxic and antiproliferative activity and also present an interesting specificity which remains to be explored. This study underlines and extends the large diversity and pharmacological potential of triterpenoid glycosides from marine sponges.
